# On Line Service Composition in the Integrated Clinical Environment for eHealth and Medical Systems

**DOI:** 10.3390/s17061333

**Published:** 2017-06-08

**Authors:** Marisol García-Valls, Imad Eddine Touahria

**Affiliations:** Department of Telematics Engineering, Universidad Carlos III de Madrid, 28911 Leganés, Spain; imad.touahria@gmail.com

**Keywords:** Integrated Clinical Environment, service composition, reconfiguration, eHealth, medical system, middleware, patient monitoring, cyber-physical system, medical service, performance

## Abstract

Medical and eHealth systems are progressively realized in the context of standardized architectures that support safety and ease the integration of the heterogeneous (and often proprietary) medical devices and sensors. The Integrated Clinical Environment (ICE) architecture appeared recently with the goal of becoming a common framework for defining the structure of the medical applications as concerns the safe integration of medical devices and sensors. ICE is simply a high level architecture that defines the functional blocks that should be part of a medical system to support interoperability. As a result, the underlying communication backbone is broadly undefined as concerns the enabling software technology (including the middleware) and associated algorithms that meet the ICE requirements of the flexible integration of medical devices and services. Supporting the on line composition of services in a medical system is also not part of ICE; however, supporting this behavior would enable flexible orchestration of functions (e.g., addition and/or removal of services and medical equipment) on the fly. iLandis one of the few software technologies that supports on line service composition and reconfiguration, ensuring time-bounded transitions across different service orchestrations; it supports the design, deployment and on line reconfiguration of applications, which this paper applies to service-based eHealth domains. This paper designs the integration between ICE architecture and iLand middleware to enhance the capabilities of ICE with on line service composition and the time-bounded reconfiguration of medical systems based on distributed services. A prototype implementation of a service-based eHealth system for the remote monitoring of patients is described; it validates the enhanced capacity of ICE to support dynamic reconfiguration of the application services. Results show that the temporal cost of the on line reconfiguration of the eHealth application is bounded, achieving a low overhead resulting from the addition of ICE compliance.

## 1. Introduction

Medical devices operate with strict safety and security standards in a rather closed manner, providing very restrictive interfaces. The reasons for this are, among others, their safety and security requirements, besides the fact that they are typically patented devices. As dictated by the non-stop technological evolution, medical systems have followed the path towards easing interoperability across vendors to facilitate the development of more powerful functions derived from the integration of heterogeneous services and equipment (servers, medical devices and sensors).

Traditionally, clinicians would continuously monitor and operate a number of devices separately. Nevertheless, for developing modern and future medical systems, it is essential to achieve interoperability among medical devices and software services. The evolution of software paradigms and implementations, and their integration with most hardware platforms, has boosted the possibilities of medical systems. Still, the medical devices that are closest to patient monitoring tend to be based on special purpose hardware, being mostly closed systems; but this trend has started to be progressively abandoned towards more interoperable standardized open approaches.

Given the critical nature of medical systems and applications, industry and suppliers have gathered around medical specifications and design approaches for realizing the interoperability goal [1]. One of the most important frameworks for medical device interoperability has been defined by the American Society for Testing and Materials (ASTM) producing the Integrated Clinical Environment (ICE, F2671-2009) [2]) , also co-sponsored by the American Society for Anesthesiology (ASA). The standard Open source Integrated Clinical Environment (OpenICE) [3] was developed by the Medical Device Plug-and-Play (MDPnP) program, and it complies to the open standard ASTM F2761, which regulates safety, security, reliability and performance of the equipment for a patient-centric integrated clinical environment. OpenICE is a distributed software platform for supporting the interconnection of network nodes such as medical devices, sensors, decision support systems or electronic medical record systems. The actual realization of ICE is not tied to any specific technological implementation. The reason for the undefinition of the underlying communication backbone of ICE is that it is a standard for achieving patient safety, defining the requirements for biomedical device integration at the point-of-care, with the goal of driving the definition of interoperability standards toward safety. Therefore, it is realizable through different underlying middleware backbones. Currently, real-world applications in the medical domain progressively adhere to the safety and security requirements of ICE; examples are the electronic health record systems, such as [4] that manages information security and on line collection of patient data.

The full realization of ICE needs to consider specific middleware technologies at different levels to enable interoperation and distribution. Precisely, the required functions are data processing and exchange formats’ definition, distribution infrastructure, flexible functional composition and transport and network protocols. The realization of the ICE framework must include middleware technologies at the levels of the bare infrastructure middleware and the enhanced middleware. Infrastructure middleware provides the basic communication paradigm for distributed communication among functional units that reside in different nodes (e.g., remote medical devices and/or server/client nodes for patient record information exchange). Enhanced middleware provides additional logic tied to a specific application domain related with either its business logic or with the behavioral requirements of the application domain. Essentially, ICE is a standard for safety interoperability across nodes, defining the roles of the different participant devices and how these should interact. Therefore, this standard must be naturally supported by (i.e., integrated with) an underlying communication bus (i.e., a distribution middleware) that provides communication functions among the participant devices; the used middleware must adjust to the non-functional properties required by the medical domains and in compliance with ICE safety requirements. This paper details the integration of ICE with a specific middleware that enables flexible reconfiguration capabilities inside ICE.

Not all middleware technologies are suitable as communication backbones in healthcare systems. For instance, in a centralized patient monitoring system that receives and displays the information about patients in their rooms/apartments, a bad decision on the selection of a middleware technology may yield latencies of over tens of seconds; also, the wrong selection may result in lack of support for flexible on line service integration. This limits the potential of on line health monitoring systems. There are different middleware possibilities to take the strong position as the communication backbone for ICE. This is the case of DDS (Data Distribution System for real-time) [5] that has been applied in OpenICE [3,6] as in a number of other distributed application environments [7]. DDS uses UDP/IP for transport and network levels and implements different levels of reliable communication, including Quality of Service (QoS) parameters for fine tuning the transmissions. Besides this and other similar proposals, additional logic is needed on top of the bare communication middleware backbone to support the flexible integration of medical services by efficient service composition: the enhanced middleware or application-specific middleware. iLand (iLand project—Reference implementation: User Guide. Sourceforge. https://sourceforge.net/projects/iland-project/) [8] is a communication middleware that provides additional logic for supporting service-based application composition and reconfiguration. iLand executes those functions in a timely way by means of its service composition and reconfiguration algorithms that employ graph theory. Therefore, integrating iLand middleware into ICE will allow ICE-based application services to reconfigure on line by modifying the number of services and/or connected devices and their interconnections. Additionally, iLand defines an architecture of an enhanced middleware that is flexible enough to be implemented over different bare communication middleware choices such as DDS, Ice (Internet Communication Engine), Corba, Java RMI, etc.; its reference implementation is built over DDS.

Therefore, integrating ICE and iLand allows on line orchestration of service-based medical applications. As an example, let us imagine a remote elderly patient monitoring scenario such as described in [9]; there, it is illustrated how services can be composed on line using iLand by means of adding new customized functions to the remote monitoring application (or by removing some services that are no longer needed). This way, the monitoring application is tailored to the specific needs of each patient. Let us imagine that, at the initial installation of the application, the essential services that it contains are: glucose level measuring and blood pressure data readings. These services may be extended by adding, on-the-fly, some additional service such as sensor-based activity detection of the patient. The software services interface with the medical devices and sensors; for instance, a blood pressure sensor can be connected through an embedded computer that runs the software that controls the service. This software provides two basic operations in its interface: read_sample and update_parameter. The first function allows collecting the information of the patient (diastolic pressure, systolic pressure and heart rate); the second function allows configuring of the device, e.g., activation of the memory functions to store patient readings for a specific period, etc. Enabling on line service addition or removal from the overall patient monitoring application would consist of connecting (or removing) the software service from the overall application or not. This can be done remotely by some specialized clinician. For example, if the clinician observes some anomaly in the patient, s/he may activate the sensor-based activity detection for the patient to obtain information on the behavioral pattern of the patient for that day and detect possible problems.

This paper describes the integration of on line composition capacities provided by iLand middleware into the ICE framework to comply with medical application requirements. The goal is to support on line flexible addition and/or removal of services to customize medical applications on-the-fly for specific patients.

For this purpose, the paper presents the characteristics of the open source middleware technology named iLand [8] that is later integrated within ICE. iLand is the result of a research project, and it was applied to different systems related to healthcare. As a result, ICE is enriched with the capacity to support flexible on line integration of medical services. Although security is a key characteristic in medical systems, it is outside of the scope of this paper. Our contribution focuses strictly on the flexible composition of the medical applications. Security aspects are included as part of ICE standard, but it is not the objective of this contribution to elaborate on them, nor include mechanisms for patient data collection and management.

The paper is structured as follows. Section 2 describes related work on middleware for medical systems. Section 3 describes the ICE framework. Section 4 describes iLand, the enhanced middleware proposal that supports decoupled timely on line service composition. Section 5 describes the integration of iLand within the ICE framework. Section 6 provides an example of a distributed service-based application distributed with iLand that performs remote patient monitoring in an elderly house; it is adapted to ICE, and reconfiguration results are presented that show the feasibility of ICE-iLand integration and the time-bounded reconfiguration. Section 7 concludes the work discussing the integration implementation.

## 2. Background

Current and future medical systems are progressively interconnected in order to flexibly exchange data among devices, departments and, most importantly, between patients and medical staff. Middleware is a fundamental software layer that enables this, and it is practically at the heart of almost every single distributed application nowadays. Middleware is characterized by abstracting the low level details of the communication protocols and the hardware characteristics of devices to programmers. This way, programmers can focus solely on the functional aspects of the application; this has a positive impact on their productivity and the application maturity level.

Among the first middleware technologies one finds RPC (Remote Procedure Call). With the years, this interaction scheme evolved towards the component-based distribution paradigm of Corba [10] and object-oriented middleware, such as Java RMI [11], among others. Message-oriented middleware also appeared on the scene like JMS (Java Messaging Service) [12]. Later, other paradigms appeared such as services with Jini [13] and data-centric publish-subscribe [5,14], or later, stream processing services [15,16]. Some middleware technologies support different communication patterns, like Ice (Internet Communication Engine) [17], which offers both, remote method invocations and asynchronous publish-subscribe through its version Ice Storm. The optimization of the design and implementation of the middleware is also a key aspect of its usability in certain domains; for example, in [18], a multicore-aware middleware is presented that exploits the characteristics of the underlying hardware platform to prioritize the service to some specific remote entities. There are countless designs of specialized middleware for individual application domains, such as the stock market [19], transport [20], healthcare [21] or remote labs for education [7]. Middleware technology is in constant progress to suit the needs of the emerging domains, such as real-time cloud computing [22].

Middleware is also key to healthcare systems for a number of reasons; just a few of them are:

- It acts as an interoperability provider, allowing information (patient records) to be communicated/transferred and formatted to the specific needs of physicians.
- It provides major flexibility across departments, as they may be interested in different patient data, which can be extracted as required from a global repository.
- It eases evolution to richer functionality in a short time such as hospitals and care centers in general, as new functions can be developed as individually-developed decoupled services.
- It allows the connection of mobile devices to the hospital network cloud, making data available to professionals anytime, anywhere.
- Patient engagement in their care could be increased as middleware can display their historic records and current monitored data in an understandable way so that they can participate in their healing process. The engagement of patients enabled by middleware requires that the middleware provide them with answers in an efficient, timely and robust manner.

One of the most frequent uses of middleware in medical systems is the automatic handling of patient records. Current practice often uses Health Information Systems (HIS) and Electronic Health Records (EHR) in an informal manner with ad hoc protocols and interoperability solutions in order to develop clinical systems. Typically, attention in these systems is paid at the pure application level where healthcare enterprise systems are put together to deliver specific clinical solutions.

Aspects such as security and safety are essential in current solutions, especially when envisioning medical cyber-physical systems, as a number of subsystems will coexist and interact through a safety/security framework. Such a framework must provide rigorous design including on line verification such as exposed in [23] and trustworthy compositional techniques to integrate devices, cooperating autonomously and in a protected mode with health information systems. For this purpose, new paradigms are appearing, such as MAP (Medical Application Platform) [1], which is a safety-critical and security-critical real-time computing platform. The most widely-adopted architectural solution nowadays is ICE (Integrated Clinical Environment) [2,24] led by the CIMITMedical Device Plug-and-Play interoperability project and later standardized.

The ICE approach defines important elements, such as the protocol stack for medical device interoperability [2]. The lower layer of this stack is DPWS (Device Profile Web Services) in charge of service discovery, interface description, messaging, event propagation and secure information transmission. On top of this pure web service communication level, a streaming dual channel transmission based on MDPWS is provided. The specific extensions for ICE devices are contained in the Basic Integrated Clinical Environment Protocol Specification (BICEPS) layer, which is above the MDPWS layer.

Below this DPWS, there is the indication of a specific technology, apart from the typical de facto usage of HTTP/TCP or UDP. However, no reference to the basic communication middleware technologies (e.g., DDS, JMS [12] or iLand [8] middleware for service oriented real-time applications, etc.) is indicated. DPWS is bound to using SOAP protocols with messages in XML, yielding to heavy communication latencies and parsing/unparsing times that may not be suitable to all domains.

Higher performance middleware alternatives to a pure DPWS backbone will yield more timely interaction between remote nodes and devices, being more suitable for the inherent temporal requirements of eHealth in contexts such as remote patient monitoring where timeliness can be a critical aspect. Message parsing activities or heavy XML message transmission may yield delays in a control loop that physically monitors the vital signs of a patient, causing errors in measurements. A number of alternative solutions for efficient transmissions must be considered for the health domain based on the specific temporal requirements of each application case.

Recently, a number of contributions on designing eHealth systems has appeared such as [25] that introduces an IoT healthcare assisted leaving design or [26] that provides a process oriented design; Ref. [27] that describes the real-time transmissions for telehealth applications; Ref. [28] that discusses security aspects on eHealth; Ref. [29] that provides a framework for integration of medical systems and distributed technology; in [30], a component model is described that allows developing medical systems for ICE-compliant applications. However, none of these contributions focuses on provisioning the ICE standard with reconfiguration and on line service composition facilities.

One of the reasons for this is that ICE specification does elaborate on the process to achieve flexible service composition; moreover, on line device plug and play is only described at a high level and restricted to standard data exchanges. There is no indication of the possibility of achieving real-time service composition. Furthermore, once the application (or a set of services) is in operation, ICE is silent on how to achieve time-bounded reconfigurations of the application due to, e.g., a change in a service or the appearance of a new service that has to be connected to the application.

## 3. The Integrated Clinical Environment

ICE is a framework for medical device interoperability; as stated in [2], it is: “a medical system designed to safely provide data acquisition, and integration and control of a heterogeneous combination of medical devices and other equipment in a high-acuity patient environment [...] intended to enable the creation of systems for innovation in patient safety, treatment efficacy, and workflow efficiency”.

**Figure 1:**
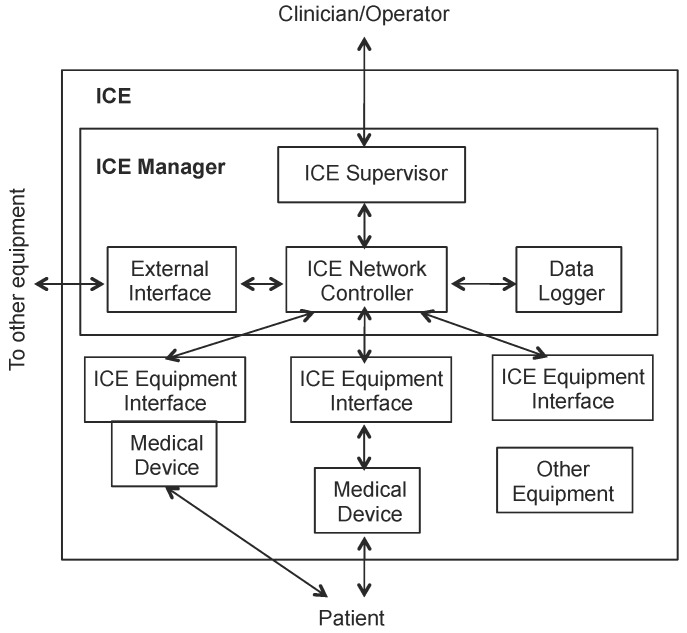
ICE functional elements (taken from ASTM Standard F2671-2009).

At the heart of the ICE idea is that medical devices must provide a compatible model aiming at interoperability (so-called ICE-compatible equipment) that is exposed through a specific interface, namely the ICE equipment interface. Figure 1 presents a high level view of the functional elements or blocks of the Integrated Clinical Environment.

The device model is a representation of the capabilities of ICE compatible equipment that includes the information needed to qualitatively and quantitatively describe, control and monitor its operation. This includes the information exposed by the manufacturer through the equipment interface, as well as the non-functional requirements according to ASTM.

The ICE network controller provides communication among ICE compatible equipment and the rest of ICE using the device model. It is in charge of ensuring that the functional capabilities, in accordance with the non-functional requirements in the device model, can be reliably delivered to the supervisor. Furthermore, it must generate alarms if the required performance cannot be delivered. An ICE network controller block manages a set of (or aggregation of) medical devices and side equipment that must provide a specified functionality meeting certain non-functional requirements. This block contains the necessary business logic according to the device model to provide the specified functional capabilities, and detecting the possible generated alarms. The ICE supervisor is a platform (both an equipment and the associated software) that provides a specific application logic (i.e., specific algorithms, etc.) and an operator interface. It ensures that the specified functional capabilities are provided according to the ICE network controller, also handling the alarms.

The ICE equipment interface is a part of an ICE-compatible equipment that provides the interface to the ICE network controller. It is typically an interface between software processes; it is not intended to be an interface between the operator and the compatible equipment.

The mission of the data logger function block is to support forensic data storage and logging that enables the detection of incidents and abnormal situations in order to initiate mitigation actions; meaningful events (operator actions, data readings, etc.) are recorded and stored and can later be analyzed to identify abnormal situations such as erroneous usage of the ICE-compatible equipment (medical devices and sensors), failure of the devices and/or of the supervisor or Ice network controller failure.

These functional blocks can be either physically separate or they can be integrated into one or more physical entities (i.e., servers, devices, etc.). The network controller is the interface where plug and play occurs. The external interface is the interface of the ICE outside of the clinical environment. According to the standard, this could be the facility backbone, public switched network or the Internet. The data logger is used to record everything within the ICE for future forensic activities, i.e., incident investigations and/or training scenarios.

Overall, ICE provides a high-level architecture mostly containing functional blocks that need to be realized by specific proposals. Such proposals must be concrete technological options containing the distribution middleware software as a basic communication backbone connecting the different medical services, with both basic communication services, as well as other enhanced logic. Most of the ICE functional blocks are wrappers that refine the basic information model provided by the device model of each equipment or device to be integrated. One of the key blocks of ICE as concerns the interaction among medical devices and services is the network controller. This element is a point of communication of a specific service or device to other services and/or devices in the medical system; it is also a point of connection to the supervisor, which contains the application logic. The following section describes a proposal of how to integrate a specific middleware technology with ICE to support both basic decoupled communication services and enhanced logic for on-the-fly service composition.

## 4. iLand Middleware: On Line Service Composition and Timely Reconfiguration

This section presents an overview of iLand, a communication middleware that is enhanced with additional intelligence to enable the time-bounded reconfiguration of distributed real-time systems based on services. This section introduces the main terminology and concepts of iLand, presenting the essential structure of the overall reconfiguration process.

### 4.1. Overview and Basic Terminology

**Figure 2:**
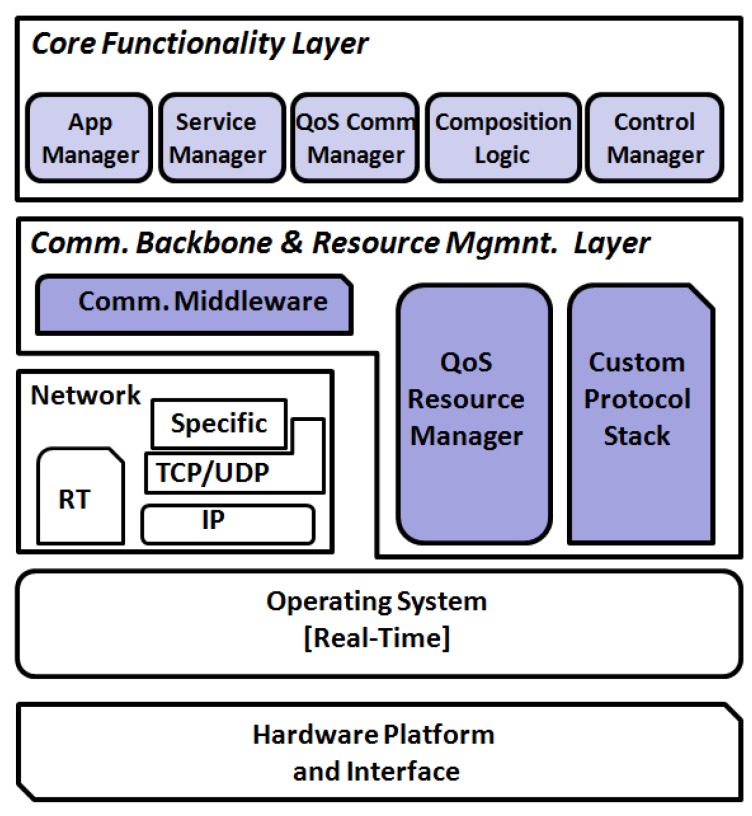
iLand middleware architecture.

iLand [8] is an open source middleware that has been applied in industrial prototypes, including medical systems. It follows the classical principles of a layered middleware design; though its architecture (shown in Figure 2) is independent of the underlying communication network protocol, the reference implementation (iLAND project. Reference implementation. Sourceforge. https://sourceforge.net/projects/iland-project/) of iLand uses a DDS backbone. iLand includes a number of enhanced functions to support dynamically reconfigurable applications based on services: light-weight services in the real-time version and web services in the soft and best effort version with QoS guarantees.

The middleware provides time-deterministic reconfiguration mechanisms (that are described in [31,32]) and service-composition functions (explained in [33,34]). Such functionality can be used in service-based applications for on line orchestration of services, supporting addition or removal of services, as well as their replacement. iLand functionality is realized by two main layers: the Core Functionality Layer (CFL) and the Communication Backbone and Resource Management Layer (CBL). CBL contains the basic infrastructure components for decoupled communications and real-time resource management, whereas CFL contains the enhanced functionality for service composition and reconfiguration. CFL components are the Service Manager (SM) that enables applications (or operators) to define and register services, i.e., self-contained stateless services; the Application Manager (AM) to define the structure of larger applications made of service sets (input as a service graph); and the Composition Logic (CL) containing time-deterministic service composition and reconfiguration algorithms.

The following is the basic key terminology of iLand:

- Service: A self-contained piece of code with well-defined interfaces that communicates with other services via message exchanges, as indicated in the Service-oriented Paradigm (SOA).
- Service implementation: It is a particular realization of a service. A given service can have different implementations that differ from one another due to their particular coding of the service interface. Two different implementations will require distinct computational resources, depending on the quality of the produced results.
- Service composition: A process by which different services are combined and orchestrated to merge as an application with global requirements such as the end to end response time. An example of this could be the connection of an oximeter service with a blood pressure service that might yield a basic daily health application for an in-house patient.
- Configuration: It is the set of software functions (i.e., services in iLand) that execute at a particular instant. A configuration is achieved by a specific service composition process. A configuration is a given set of service implementations.
- Reconfiguration: A transition from the current configuration to a new (i.e., target) configuration. This reflects a change in the application functions. An example might be the addition of a third temperature sensing service to the former basic daily health application.
- Time bounded reconfiguration: It is a reconfiguration process performed within a guaranteed maximum amount of time, i.e., deadline. It includes a time-bounded service composition algorithm [31].
- Real-time pruning and low-complexity reconfiguration: A reconfiguration process may involve the exploration of all possible combinations of configurations that could yield a high-complexity process. iLand designed a low-complexity reconfiguration process [33,35] where the initial space of solutions is summarized as a representative subset of the original graph. Therefore, only this subset is explored to check if any new tentative configuration meets the specification and requirements of the system.

### 4.2. Service Composition and Reconfiguration

**Figure 3:**
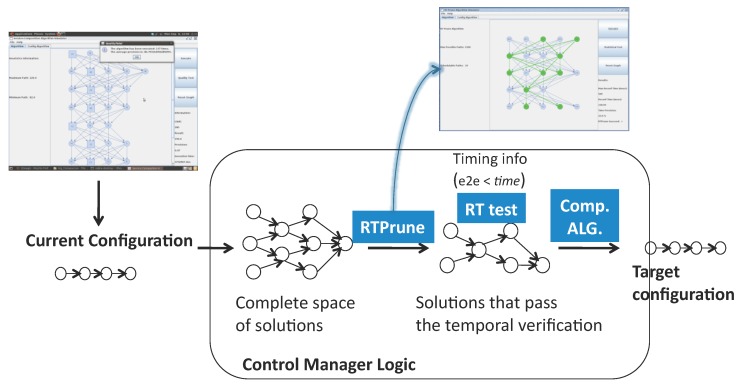
Reconfiguration process coordinated by the control manager entity of iLand. Different modeling tools aid the off line fine-tuning of the system.

Figure 3 illustrates the reconfiguration process for distributed real-time applications that consists of on line modifications of the structure and connection of services in a time-bounded manner.

**Figure 4:**
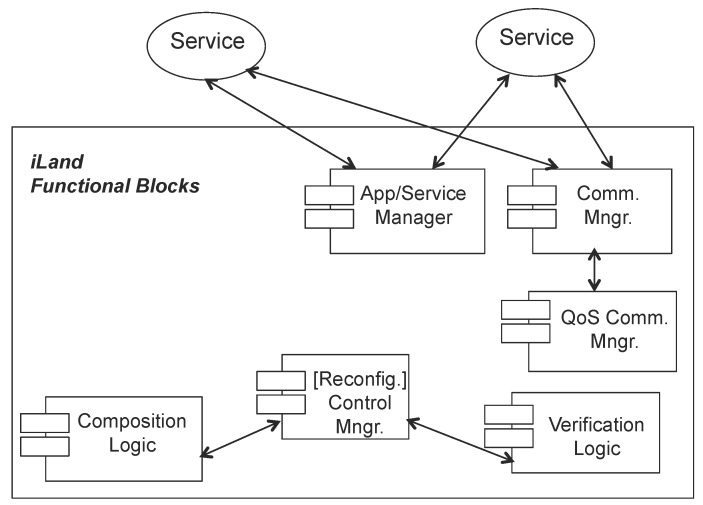
Enhanced core functionality layer with explicit illustration of the verification component.

Distributed real-time systems can be reconfigured with iLand middleware ensuring the timeliness of the transition from the current configuration to a new one. The reconfiguration logic is contained inside the control manager component. Configurations are expressed as service graphs with end to end timing properties, and a set of restrictions are imposed to achieve time-bounded transitions on line:

- Off line exploration of the space of solutions: Prior to execution, the system design is fine-tuned for efficiency and timeliness. Two tools, generalized BTEA for timely service composition [31] and Real-Time Prune (explained in [33,35]) for complexity reduction, are used for the assessment of whether the timing behavior of the reconfiguration is valid for the specific designed application.
- Reliable communications: The communication time between distributed services assumes that the underlying communication transport is reliable and there is a real-time network with guaranteed message scheduling.
- Centralized verification of the configurations: The iLand reference implementation includes a single verification entity that has the view of the whole system (see Figure 4). The verification of an application is essential for the cyber-physical domain to prove that the configurations satisfy the requirements at all times. iLand includes a temporal verification component that applies utilization-based schedulability analysis to check the temporal behavior of the applications.
- Centralized reconfiguration coordination: There is a single entity that coordinates the reconfiguration process, mandating over the execution of the reconfiguration phases that are required for the transition between the current and the target configurations.
- Emergency configuration: As a mechanism to achieve fault tolerance, iLand has a back-up configuration that satisfies the system specification at all times. In the event that the reconfiguration process does not find a target configuration that satisfies the system specification, the emergency configuration is applied.

The interface between the application level (including an operator) and iLAND is at the level of the application/service manager and communication manager components. Applications or an operator can register services and configure specific applications with the App/Service manager; the communication among services is supported by the functions of the communication manager component.

### 4.3. iLand Ease of Use and Application Development

The experiences gained with iLand have shown that the middleware is easy to deploy and also facilitates the design and execution of applications. For applications designed as decoupled stateless services, the middleware provides a plug and play approach for application reconfiguration and service orchestration, i.e., new services may be added, and running services can be deleted or replaced. The design of the application is indicated in the form of a configuration file or using a front-end tool.

Following, the path to having an application up and running on iLand middleware is explained:

- The initial step is to model the application as a Service-Oriented Architecture (SOA). Services must provide the interface specified by iLand API (iLand project—Reference implementation: User Guide. Sourceforge. https://sourceforge.net/projects/iland-project/). In the interface, a service specifies a functional part and the non-functional part [36]. The main non-functional parameters relate to the time requirements. Once in execution, dynamic modifications of the application will be supported (i.e., reconfiguration). This means that an application will be able to either replace, stop or launch a given service, therefore modifying its service graph. Moreover, this reconfiguration is supported in a timely manner, i.e., an upper bound for the reconfiguration time is guaranteed.
- Physically locate each service at a given remote node in the network. Both connections through Internet protocols and custom real-time network communications are supported.
- Each physical node in an iLand network will have to install the iLand middleware libraries. Different profiles are available for iLand middleware: star (with full functionality), planet (for nodes without the reconfiguration coordination modules) and satellite (for nodes with limited capabilities in the iLand connected services; these correspond to physical sensors, and their functions are to sample data from the physical system (e.g., patients, environment, etc.) and forward them to a planet or star configuration and also to receive commands from a planet node).
- The star configuration supports the design of the specific application SOA in both a configuration file or with a model-driven tool front-end. Furthermore, it allows launching the services. Once services are in execution, these are automatically connected as specified.
- A list of reconfiguration events can also be specified in the configuration file. Apart from these, the logic of the services also allows detecting reconfiguration triggers (e.g., a value of some biometric parameter is beyond the specified threshold so a different biometric service must also be activated, and an alarm should be signaled in the control center).

Once in execution (and upon the triggering of the reconfiguration events), iLand automatically handles the system structure and makes timely transitions from the current configuration to the new one. iLand implements a constant monitoring activity to detect situations requiring a reconfiguration of the system. This logic has proven to be efficient with little overhead, as well as the reconfiguration logic that performs a previous selection of the target system configuration.

## 5. On Line Service Composition in ICE

Based on the previously presented ICE and iLand architecture, this section presents their integration. As a result of the integration, ICE embeds additional logic to: (1) support flexible communication among the software services and the medical devices and to (2) provide reconfiguration capabilities to applications for on line integration (or removal) of services.

ICE is simply a generic architecture to design medical devices and services that can be easily interconnected. However, it only defines the general view of the basic components to interconnect devices among themselves and with a clinician or operator. Consequently, ICE does require the integration of specific software technology designs to: (1) enable flexible communications among devices; and (2) additional logic for improved behavior, such as the on line composition of services.

By integrating iLand into ICE, the capacities of the latter are increased by offering flexible on line service plug and play; as iLand supports on line application definition based on service graphs with end-to-end time requirements. This is a clear improvement for the ICE architecture as applications can be defined and modified on line by operators according to the specific changing patient needs.

The iLand architecture is based on the classical definition of middleware architecture [37], later revisited for cyber-physical systems in the Oma-cy architecture [38]. In these, there is a clear layered view based on basic operating system services, infrastructure middleware, distribution middleware and application-specific middleware. To comply with the high-level abstract architecture definition of ICE, the integration is provided following the architectural presentation of ICE, i.e., showing the functional blocks, as illustrated in Figure 1.

### 5.1. Reconfiguration Logic Principles

Following, it is indicated how the interaction between ICE and iLand occurs for the on line service registry and application reconfiguration. In this case, there is an application running made of two devices or services that are connected as a directed graph of the form Device${}_{1}\to$ Device${}_{2}$. This application needs to be modified by adding a new service (Device${}_{3}$) that is not yet registered in the system.

**Figure 5:**
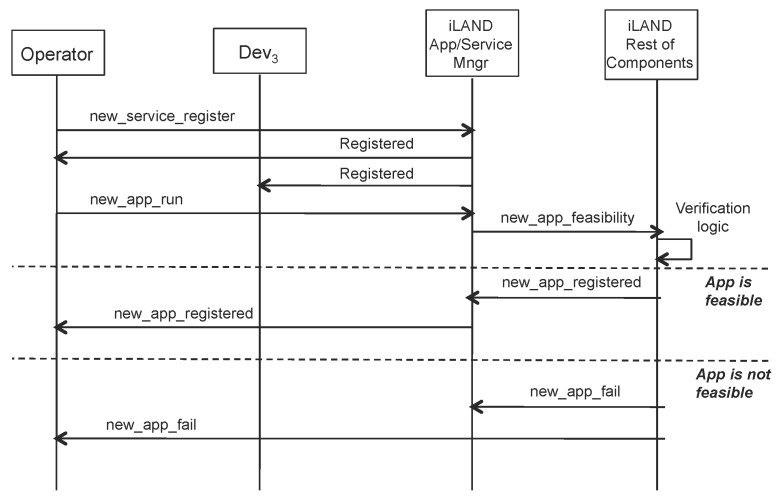
ICE-iLand device and operator interaction for service registry and creation of a new application.

Figure 5 presents the interaction between the different actors in the new context. iLand supports that operators request the registry of a new service that is activated in the system, but also it supports that a service automatically initiates this request. If an operator wishes to launch a new application, the operator will first enter the concrete service graph with specific interconnections among services. A request to register a service (new_service_register) can then be granted. However, requests to run a new application (or to reconfigure an existing one) have to be checked for feasibility and correctness. The verification logic of iLand is run to make sure that the new application execution will comply with the requirements expressed in the invocation to new_app_run and that the rest of the already running services will not suffer interference. If the requested new application is feasible, then the control manager component of iLand will execute the reconfiguration protocol and ensure a timely transition to the new configuration.

### 5.2. Integration

There are two ways to perform this integration. A first approach is to include specific iLand components that provide on line service composition as functional blocks. This option is, however, invasive with respect to the ICE architecture, and it would require modifications to the standard high-level definition of ICE. A second approach is to preserve the independence of ICE functional blocks, including only an additional block that contains the minimum iLand services that guarantee the time-bounded service communication, composition and reconfiguration. This second approach is adopted as it minimizes invasion and coupling among ICE and iLand while providing the benefits of both. The integration of the reconfiguration logic does not affect the medical devices’ operation. Medical devices are connected to the distributed medical system through a gateway: the ICE equipment interface. This interface interacts with the ICE network controller that is the entry point to the ICE manager part, in the sphere of: the supervisor, the external interface and the data logger. iLand is located in the ICE manager part, and it only interacts with the ICE supervisor and with the external interface.

**Figure 6:**
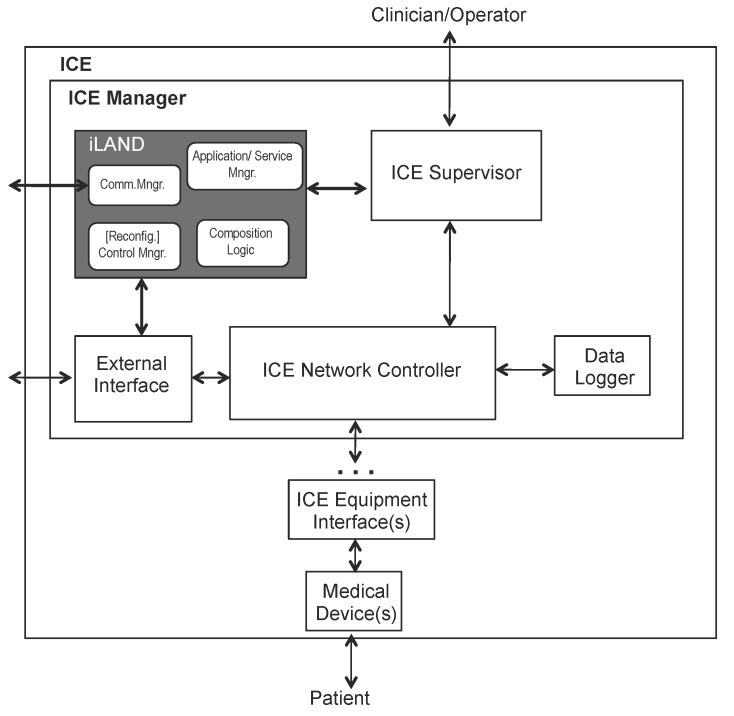
Extending ICE for supporting on line flexible service composition and reconfiguration: iLand-ICE integration.

Figure 6 presents the integration. Only the basic components of iLand layers (Core Functionality Layer (CFL) and Communications Backbone and resource management Layer (CBL)) are presented in the integration architecture. These are provided in a compact iLand module that contains:

- Communication Manager (Comm. Mngr): It includes the main infrastructure middleware functions that provides real-time communications (through a custom protocol stack) or QoS communication (through standard transport protocols). If the underlying communication backbone supports QoS parameters (such as CORBAor, even more, DDS), iLand offers the possibility of setting such parameters.
- One or more medical device can be managed under the same ICE supervisor to provide a specific functionality. These medical devices can be provided to other applications as services, e.g., oximeter data reads provided as a software service to a nurse control center set. Therefore, the ICE supervisor needs to integrate some additional logic to keep the structure of applications and services interconnection; this is enabled by the application/service manager component. In iLand CFL, there are two separate components for managing applications and services. The Service Manager (SM) allows registering and eliminating individual services in the system, specifying their functional and non-functional properties and interfaces. The Application Manager (AM) component supports the definition of applications that are made of aggregations of services and that have global functional and non-functional properties; these are specified as service graphs. For simplicity, the interfaces to these two components are provided in a unified way in this integration of iLand-ICE.
- For supporting on line composition of the services, ICE needs to integrate both the service composition logic (composition logic component) to provide on line medical services integration, as well as the logic that coordinates the actual selection of services ((Reconfig.) control manager component).

Vendors guarantee device interoperability by providing a standard interface for each device: the ICE equipment interface. Compatibility across the functions of different devices is provided at the application level, within the ICE supervisor that is in charge of collecting, analyzing and displaying the data from devices either individually or in an integrated way; this will depend on the specific logic of the application that is running, e.g., the ICE supervisor may be executing an application that simply displays the different data collected by the medical devices that monitor the patient vital conditions; or it may also display inferred data derived from the cross-processing of the monitored data.

## 6. An Example of Services Integration in a Medical Environment

This section presents a monitoring system for an elderly house that is based on an initial implementation on iLand middleware. In this section, it is explained how it has been adapted to the Integrated Clinical Environment (ICE) to enhance its interoperability with devices following the ICE architecture.

**Figure 7:**
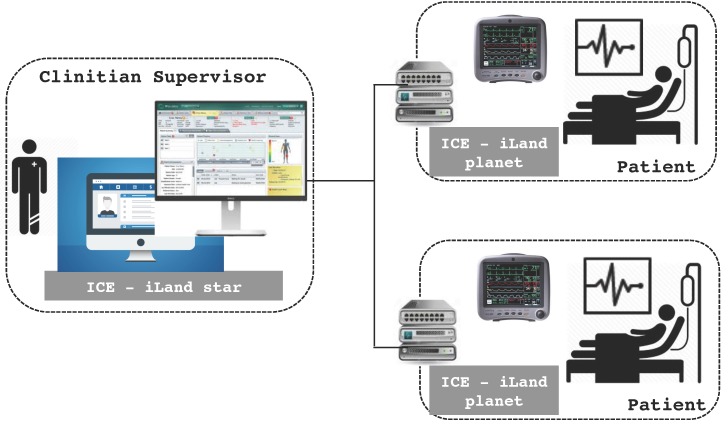
Elderly house real-time monitoring system.

In this specific example, patients may be in severe physical conditions; therefore, they need to be monitored in real time. They are located in a centralized building (elderly house), and each patient has an individual room specially configured according to the patient’s health requirements. This means that the devices and sensors across rooms are different and, also for a specific patient, these devices can vary over time according to the person’s needs. Figure 7 presents the general overview of the system. Patient’s individual spaces are equipped with specialized medical equipment integrated in the room. Sensors (that are intermediated by means of embedded computers like Raspberry Pi [39]) perform vital sign sampling (e.g., oximeter and pulse meter); once sensor data are captured, these data are processed, logged and transmitted to a supervisor node in the same building with the continuous presence of clinicians.

Each room has a service patient manager (or room manager) and a set of medical sensors to monitor the patient health conditions. The room manager is a front-end monitoring and decision making system that performs basic analysis of the patient’s monitored data. The medical equipment subsystem is simulated, and the gathered data are synthetically generated to test monitoring algorithms with physical parameters analysis. The room manager is an embedded system with a basic interface, integrated with the room. The clinician supervisor server (or control center) is equipped with iLand middleware and a database connection.

Time requirements are specified in this system as there are different criticality levels, some being highly critical. The periodicity of the medical patient monitoring samples range from 1 s to 1 min, according to the patient health conditions. Data logs to the control center are stored in 1-s periods. For any detected alarm, the patient manager must trigger the clinical supervisor within a specified deadline in the range of 500 ms to 1 s.

Different reconfiguration scenarios are possible. Reconfigurations are triggered by the occurrence of some specific events (reconfiguration triggers), and they start a process to perform a new service composition in the system. The occurrence of a reconfiguration trigger may be the arrival of a new patient; room adaptation according to patient health conditions; and alarm event detection (due to, e.g., a sensor detecting values higher than a safe threshold for a specific patient’s vital sign). Therefore, it is evidenced that a reconfiguration trigger requires a modification in the set of services that are active in the system at the moment that it occurs.

**Figure 8:**
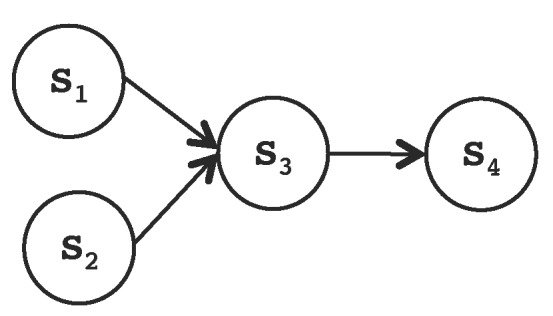
Initial scenario for a medical service-based application.

Figure 8 shows a medical application example. Applications are structured as a set of services; for this reason, applications are also referred to as SOA, which are a set of services that cooperate and exchange information and events to perform a common goal. The example application has the following services:

- data collection services ($S_{1}$ and $S_{2}$) that operate two sensors that sample two corresponding vital signs from a patient;
- data analysis service ($S_{3}$) that performs initial processing of the collected samples creating basic data logs with the measured data from the monitored patient;
- log sending service ($S_{4}$) that forwards the logs to the appropriate location in the system for storage.

**Figure 9:**
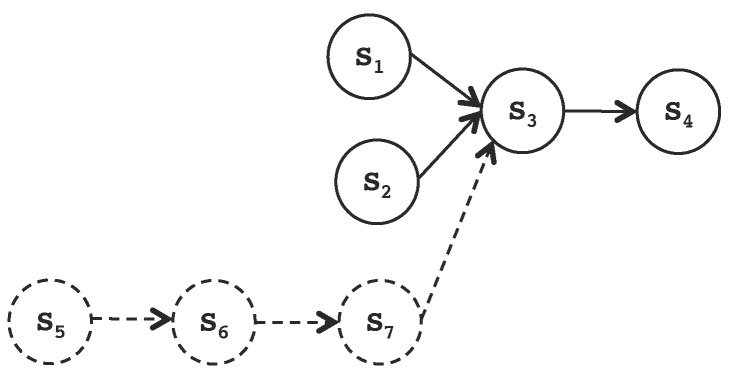
New structure of the service -based application after the reconfiguration trigger that yields a new service composition (the new services are indicated with dashed lines).

Figure 9 shows the structure of the application as a result of a reconfiguration trigger to add new equipment to the room. A new sensor will be added to monitor some specific vital sign of the patient. This implies adding three additional services:

- read new room configuration service ($S_{5}$) that parses the configuration file created by the clinician (on site or remotely);
- device control service ($S_{6}$) that is service that effectively connects the new device or sensor as part of the service set;
- collect data service ($S_{7}$) that captures the data sampled by the new sensor.

**Figure 10:**
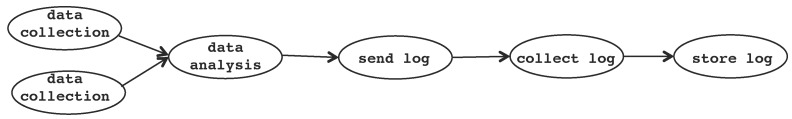
Data collection services: patient data collection, log creation and store log.

Let us now introduce an example medical application for remote patient monitoring. The site where each patient is located is configured ad hoc for the patient; this includes instantiating the required services to operate the sensors, collect sampled data, store them and transmit them to the clinician site. For each patient room, two service-based applications are instantiated (see Figure 10): (i) data analysis; and (ii) patient monitoring. The set of services for the application data analysis is made of the following services (for the case of two medical sensors):

- data collection services obtain the data sampled by the medical sensors; each sensor has an individual service that collects its sampled data;
- data analysis fuses the data and reasons about the profile of the patient in order to determine the current health conditions of the patient and commits the result to the local storage;
- data logs format the sampled data and the obtained health status of the patient and send them to the clinical supervisor;
- transfer logs execute at the clinical supervisor side by retrieving the logs sent by the room managers and forwards them to a storage server;
- store logs store the monitored data of the patients in persistent storage.

**Figure 11:**
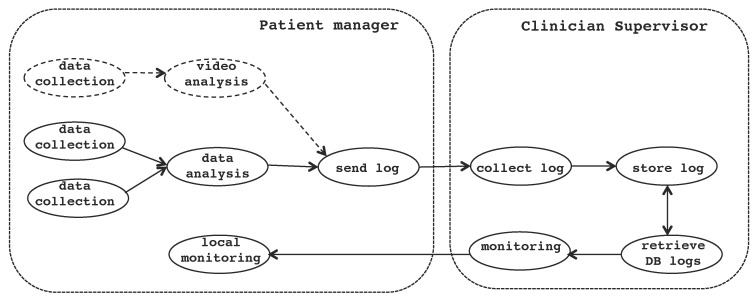
Physical service deployment, including a reconfiguration scenario for the activation of video monitoring.

The set of services of the patient monitorization application (see Figure 11) are: (i) read database to retrieve stored logs from the data base; (ii) supervision that analyses the logs (patient data) on the control center to determine the patient health conditions; and (iii) local supervision is run by the room manager and performs on-site local results analysis.

An additional SOA performs log data display to medical staff in the control center. The log data display SOA has two services: read DB (for interfacing to the database) and display for handling data formatting and display.

This design allows handling each room individually while preserving continuous operation through a single end point for control. As an example, upon arrival of a new patient, it is possible to instantiate the two SOAs needed for data collection and supervision/monitoring in this room to configure them without affecting the rest of the system. Likewise, reconfiguration only concerns the data collection SOA of a single room and not the whole system. Figure 11 shows the relation between the mentioned SOAs.

### 6.1. Prototype Execution with ICE-iLand

A similar scenario to the one proposed above was originally implemented with bare iLand [9]. Here, it is adapted to become an ICE-aware medical system; it has been derived naturally as will be explained in what follows. The mapping between the service-based application provided by iLand and the ICE architecture will now be presented.

**Figure 12:**
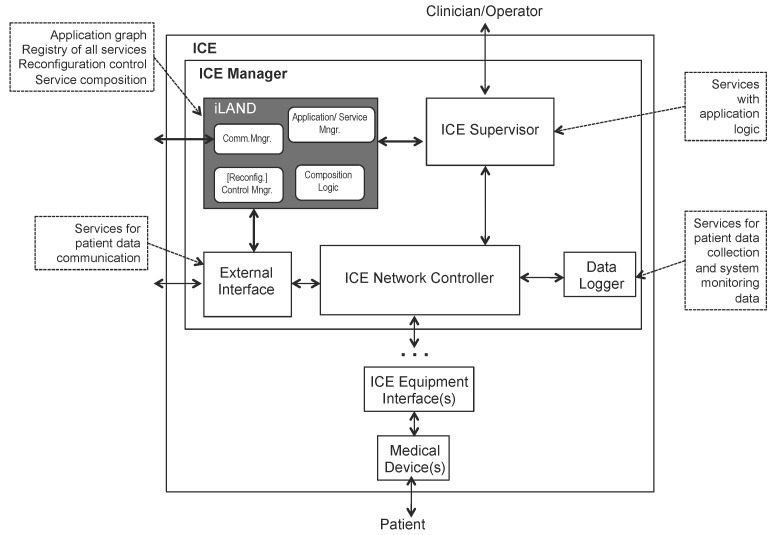
Key integration points for iLand-enabled applications and ICE.

Figure 12 presents the basic ideas behind the integration of a service-based iLand application with ICE. The main ideas in this integration are the following:

- Preserving the service-based design, as it supports decoupled application development and enhances system flexibility; for this purpose, all services must be registered in the iLand component that will keep the overall view of the medical application (i.e., the list of all existing services and the application graph).
- Mapping all functional blocks of ICE to the specific services that are present in the example; the service-based design and iLand low-complexity architecture provides a natural way to perform this mapping.

Following, the specific mappings between the elderly house application design based on services and ICE architecture are presented:

- Each sensor in the example is a medical device that runs an ICE equipment interface according to its specified device model. These devices perform autonomous sampling of patient data that is restricted to data sensing and reading simulations that provide the monitored data at a predefined frequency.
- Data collection services are the software interface of the sensors. Measure data services obtain the patient monitored data by the sensors via the ICE equipment interface. Sensors are exported as services in the iLand application; this way, each sensor can be accessed individually and connected on line to build a specific application. Therefore, iLand services are simply wrappers to the sensors’ functionality.
- Collect log and retrieve DB logs are services that correspond to the ICE data logger functional block; again, they only require providing wrappers to the data logger functionality. The services data are exported to the data logger via an interface to guarantee abstraction independence between the services implementation and the functional ICE blocks. In this way, the iLand application structure information, the reconfiguration logic and registry/deletion of services is kept independent from the ICE functional blocks.
- Services read new room configuration and device control control each of the services implemented for sampling data (i.e., measuring data from patients). Every time a new device or sensor is installed, these services are uploaded to make it usable in the iLand system.
- The room manager module contains a number of services that are mapped to the ICE supervisor. This service is, precisely, the local monitoring. Again, this iLand service is exported to the ICE supervisor via an interface to guarantee abstraction independence between the services implementation and the functional ICE blocks.
- Send log service is mapped to the ICE external interface that provides communication to other devices that are, in this case, used for sending the patient data to another system, such as a server, etc.

### 6.2. Implementation Results

This section presents the practical implementation of this example. Initially, the implementation over iLand is presented; later, the example is ported to comply with the ICE architectural framework. This section shows the feasibility of integrating an iLand-enabled distributed service-oriented medical application that supports reconfiguration into an ICE architecture, providing experimental results that show the reconfiguration time achieved with iLand for the timely delivery of alarms.

Firstly, a real-time situation is presented with low latency requirements. It is considered that during the normal operation of the system (i.e., when the patient vital data are being monitored), an alarm may occur. The reasons for this may be the detection of abnormal sensed values that would require immediate signaling.

iLand supports a number of underlying communication backbones using both Internet transports and/or custom real-time network protocols. In this situation, the underlying communication protocol needs to be highly reliable and timely; data cannot be lost nor suffer unbounded delays. For this system, iLand middleware was adapted to time-triggered communication, which enables real-time schedulability analysis in the verification component. The time-triggered protocol [40] was analyzed to ensure that it provides sufficient flexibility for asynchronous event transmission over Ethernet, like alarms. The usual approach is to fix periodic time slots for alarm events. iLand middleware was ported to run a time-triggered protocol with scheduled time-slots that guarantees real-time asynchronous traffic transmission such as alarms.

The goal of this initial experiment is to measure the reconfiguration time as the time to forward an alarm to the room manager and to the clinician terminal when an alarm has been detected during the normal operation of the system. Results are shown in Table 1 for different ECs or elementary cycles, precisely for 10-ms and 50-ms ECs for the time-triggered communication. Stability is evidenced in the resulting times, showing efficiency and real-time transmission deadline preservation for all cases. The example shows that the middleware provides stable response times and guarantees an upper bound on the alarm handling.

In the following experiment, a reconfiguration use case is provided as the result of a new service being registered in the system (similarly to the example shown in Figure 11). Two situations are explored: (1) the on line registration of a new medical device, an oximeter sensor, that is configured for a specific patient; and (2) the detection of an alarm that requires launching an already registered service that is currently inactive. The goal of this new setting is to shown that these situations are handled in the ICE-compliant iLand-based distributed service application, guaranteeing a bounded reconfiguration time and without interfering with the system operation.

Table 2 summarizes the results obtained for the first situation. Overhead cost caused by the adaptation to ICE (compared to [8]) is less than 9.4% for the worst case time and around 4.93% for the average case.

For the second situation, experiments were carried out to obtain the reconfiguration time, i.e., the time taken by the system to launch the video surveillance service since the instant that the alarm is detected. Experiments were conducted over 1000 reconfiguration events.

In this scenario, the distributed application contains services to monitor patient data (data collection), to obtain patient data logs (data analysis) and remote video surveillance of the patient (video analysis). The reconfiguration is triggered when an alarm is detected. The figure shows the time to start the video surveillance service that is physically linked to an IP camera connected to its controlling node via an Ethernet interface. A UDP transport is used for iLand over DDS. It is shown that the worst case reconfiguration time never exceeds 10.521 ms for this ICE-compliant iLand-based service system. The overhead for an ICE application that is able to perform on line service composition is only 4.93% for the average case and 9.4% for the worst case. Therefore, it is shown that the implementation is consistent with the expectations: (1) the integration continues to preserve time-bounded reconfiguration, and (2) the overhead incurred by the integration with ICE is measurable.

## 7. Conclusions

This paper has presented a contribution for supporting dynamic reconfiguration in ICE. The approach is based on the usage of iLand middleware that provides time-bounded reconfiguration of distributed applications based on the service-oriented paradigm. This approach is supported by the proven flexibility of iLand in different real application domains, including medical environments.

The paper has presented the characteristics of the Integrated Clinical Environment, indicating its gaps with reference to the specific technologies and middleware for enabling decoupled communication and on line flexibility for service deployment. iLand middleware has been presented as a choice for the flexible development of distributed systems based on medical services. This integration of iLand in ICE has been done for two fundamental reasons, which are explained as follows.

On the one hand, the current trend to provide technologies that enable flexible and interoperable services requires that contributions meet the safety guarantees that are fundamental in the medical domains. The latter is the main purpose and goal of the Integrated Clinical Environment.

On the other hand, ICE is an abstract architecture that defines general functional blocks to define safety-compliant architectures for medical systems. However, the initial definition of ICE focuses on medical device interoperability, and it is silent about functional reconfigurations. The latter is precisely covered by iLand middleware. The interaction paradigm of iLand is a decoupled one, therefore supporting decoupled integration of services that can be either local in the same node or distributed among different nodes in different network segments. iLand middleware has a modular architecture enabling easy porting to different underlying communication middleware technologies; these range from the most conventional and de facto standards, such as DDS, Corba, RMI, Ada DSA, or lower level networking protocols operating at the medium access layer such as time-triggered ones. The reconfiguration logic of iLand is based, on the one hand, on graph algorithms for representing applications and the relation among their constituent services; on the other hand, it is based on search techniques that target timely decision making and a set of steps to guarantee the reconfiguration of a whole system based on services.

Therefore, there is a benefit in integrating both worlds so that service-oriented applications with real-time guarantees can be provided in a way that is compliant with ICE.

The paper has provided the mappings between the services of a medical application based on iLand and the ICE architecture. The fact that iLand uses a decoupled service-oriented interaction scheme is an advantage for its integration within ICE. The paper has shown the mapping of the different services that are registered in the iLand middleware to the different functional blocks of ICE. A specific example application has been implemented for the remote monitoring of elderly patients, showing the timeliness of the middleware, and the bounded reconfiguration times achieved in two different reconfiguration situations: on line addition of new sensors and on line activation of existing services.

Reconfigurations are guaranteed to be completed in bounded time: the modification of a service (replacing a specific implementation) or an SOA (replacement of the service graph) is achieved within the specified deadline, showing the time measurements performed for the elderly house case; time measures confirm the real-time operation of the system and the stability of the reconfiguration logic within an ICE-enabled framework.

## Figures and Tables

**Table 1:** Alarm handling time (ms).

EC	Average Handling Time	Maximum Handling Time
10	1.086	1.098
50	5.368	5.402

**Table 2:** On-line service registration time (ms).

	iLand	ICE-iLand	Overhead (%)
**Max.**	9.578	10.521	9.4
**Min.**	6.530	7.024	7.57
**Avg.**	7.753	8.082	4.93

## Abbreviations

ICE	Integrated Clinical Environment
MDPnP	Medical Device Plug and Play
OpenICE	Open implementation of ICE
BICEPS	Basic Integrated Clinical Environment Protocol Specification
HIS	Health Information Systems
EHR	Electronic Health Record
DPWS	Device Profile Web Services
MAP	Medical Application Platforms
DDS	Data Distribution Service
JMS	Java Messaging Service
RMI	Remote Method Invocation
SOA	Service-Oriented Architecture
BTEA	Bounded Time Execution Algorithm
RTPrune	Real-Time Prune
QoS	Quality of Service
CFL	Core Functionality Layer
CBL	Communication Backbone and resource management Layer
CL	Control Logic
SM	Service Manager
AM	Application Manager
SOAP	Simple Object Access Protocol

## References

[1] Hatcliff J., King A., Lee I., MacDonald A., Fernando A., Robkin M., Vasserman E., Wininger S., Goldman J.M. Rationale and architecture principles for medical appliction platforms. Proceedings of the 3rd IEEE/ACM Conference on Cyber-Physical Systems (ICCPS).

[2] ASTM International (2009). ASTM F2761—Medical Devices and Medical Systems—Essential Safety Requirements for Equipment Comprising the Patient-Centric Integrated Clinical Environment (ICE). http://www.astm.org/Standards/F2761.htm.

[3] Plourde J., Arney D., Goldman J. M. OpenICE: An open, interoperable platform for medical cyber-physical systems. Proceedings of the 2014 ACM/IEEE International Conference on Cyber-Physical Systems (ICCPS).

[4] Ice Health Systems Electronic Health Record, v5.18.1.0.

[5] Object Management Group A Data Distribution Service for Real-time Systems Version 1.4. http://www.omg.org/spec/DDS/.

[6] Goldman J.M. (2008). Medical Devices and Medical Systems-Essential Safety Requirements for Equipment Comprising the Patient-Centric Integrated Clinical Environment (ICE)-Part 1: General Requirements and Conceptual Model.

[7] García-Valls M., Basanta Val P. (2013). Usage of DDS data-centric paradigm for remote monitoring and control laboratories. IEEE Trans. Ind. Inform..

[8] García-Valls M., Lopez I.R., Villar L.F. (2013). iLand: An enhanced middleware for real-time reconfiguration of service oriented distributed real-time systems. IEEE Trans. Ind. Inform..

[9] García-Valls M., Herrasti N., Jouvray C., Armentia A. (2017). Flexible and timely on-line integration of medical services using iLand middleware. ACM SIGBED Rev..

[10] Object Management Group: The Common Object Request Broker Architecture and Specification, Version 3.3. http://www.omg.org/spec/CORBA/3.3.

[11] Sun Microsystems: Java^TM^ Remote Method Invocation API. http://docs.oracle.com/javase/7/docs/technotes/guides/rmi/.

[12] Deakin N. (2013). Java Community Process—JSR 343: Java^TM^ Message Service 2.0.

[13] Apache Software Foundation (2013). Jini^TM^ Network Technologies Specification. https://river.apache.org/doc/spec-index.html.

[14] Information Technology Task Force (ITTF), ISO/IEC OASIS AMQP1.0—Advanced Message Queuing Protocol (AMQP), v1.0 Specification; ISO/IEC 19464: 2014. http://docs.oasis-open.org/amqp/core/v1.0/os/amqp-core-overview-v1.0-os.html.

[15] Stephens R. (1997). A survey of stream processing. Acta Inform..

[16] Su X., Swart G., Goetz B., Oliver B., Sandoz P. (2014). Changing engines in midstream: A Java stream computational model for big data processing. Proc. VLDB Endow..

[17] ZeroC Inc. (2016). The Internet Communications Engine v 3.6. http://www.zeroc.com/ice.html.

[18] García-Valls M., Calva-Urrego C. Improving service time with a multicore aware middleware. Proceedings of the 32nd ACM/SIGAPP Symposium on Applied Computing (SAC).

[19] Oliveira J., Pereira J. Experience with a middleware infrastructure for service oriented financial applications. Proceedings of the 28th ACM Symposium on Applied Computing (SAC).

[20] Martins R., Lopes L., Silva F., Narasimhan P. Stheno, a real-time fault-tolerant P2P middleware platform for light-train systems. Proceedings of the 28th ACM Symposium on Applied Computing (SAC).

[21] Arney D., Plourde J., Schrenker R., Mattegunta P., Whitehead S.F., Goldman J.M. (2014). Design Pillars for Medical Cyber-Physical System Middleware. Medical Cyber Physical Systems—Medical Device Interoperability, Safety, and Security Assurance (MCPS 2014).

[22] García Valls M., Cucinotta T., Lu C. (2014). Challenges in real-time virtualization and predictable cloud computing. J. Syst. Archit..

[23] Bersani M.M., García-Valls M. (2017). On-line verification in cyber physical systems: Practical bounds for meaningful temporal costs. J. Softw. Evol. Process.

[24] Gregorczyk D., Fisher S., Busshaus T., Schlichting S., Pöhlsen S. (2014). An Architecture for Distributed Systems of Medical Devices in High Acuity Environments.

[25] Corno F., De Russis L., Roffarello A.M. A Healthcare Support System for Assisted Living Facilities: An IoT Solution. Proceedings of the of 40th IEEE Annual Computer Software and Applications Conference (COMPSAC).

[26] Beyer M., Kuhn K.A., Meiler C., Jablonski S., Lenz R. Towards a flexible, process-oriented IT architecture for an integrated healthcare network. Proceedings of the 2004 ACM symposium on Applied computing.

[27] Wang J., Qiu M., Guo B. (2017). Enabling real-time information service on telehealth system over cloud-based big data platform. J. Syst. Archit..

[28] Wang J., Abid H., Lee S., Shu L., Xia F. (2011). A Secured Health Care Application Architecture for Cyber-Physical Systems. Control Eng. Appl. Inform..

[29] Gregorczyk D., Fischer S., Busshaus T., Schlichting S., Pöhlsen S. An Approach to Integrate Distributed Systems of Medical Devices in High Acuity Environments. Proceedings of the 5th Workshop on Medical Cyber-Physical Systems, OpenAccess Series in Informatics (OASIcs).

[30] Touahria I.E., García-Valls M., Khababa A. An ICE compliant component model for medical systems development. Proceedings of the 41st IEEE Conference on Computers, Software, and Applications (COMPSAC).

[31] García-Valls M., Basanta-Val P. (2013). A real-time perspective of service composition: Key concepts and some contributions. J. Syst. Archit. Embed. Syst. Des..

[32] García-Valls M., Basanta-Val P. (2014). Comparative analysis of two different middleware approaches for reconfiguration of distributed real-time systems. J. Syst. Archit. Embed. Syst. Des..

[33] García-Valls M., Uriol-Resuela P., Ibánez-Vázquez F., Basanta-Val P. (2014). Low complexity reconfiguration for data-intensive service-oriented applications. Future Gener. Comput. Syst..

[34] García-Valls M., Alonso A., de la Puente J.A. (2012). A Dual-Band Priority Assignment Algorithm for QoS Resource Management. Future Gener. Comput. Syst..

[35] García-Valls M., Basanta-Val P., Estévez-Ayres I. (2011). Real-time reconfiguration in multimedia embedded systems. IEEE Trans. Consum. Electron..

[36] García Valls M., Basanta-Val P., Marcos M., Estévez E. (2014). A bi-dimensional QoS model for SOA and real-time middleware. Comput. Syst. Sci. Eng..

[37] Schantz R., Schmidt D., Masters M.W., Cross J.K., Martin L., Sharp D.C., Dipalma L.P. (2002). Towards Adaptive and Reflective Middleware for Network-Centric Combat Systems.

[38] García Valls M., Baldoni R. Adaptive middleware design for CPS: Considerations on the OS, resource managers, and the network run-time. Proceedings of the 14th Workshop on Adaptive and Reflective Middleware (ARM).

[39] García-Valls M., Ampuero-Calleja J., Ferreira L.L. Integration of Data Distribution Service and Raspberry Pi. Proceedings of the 12th International Conference on Green, Pervasive and Cloud Computing (GPC).

[40] Kopetz H., Bauer G. (2003). The time-triggered architecture. Proc. IEEE.

